# Single-Walled Carbon Nanotube-Assisted Antibiotic Delivery and Imaging in *S*. *epidermidis* Strains Addressing Antibiotic Resistance

**DOI:** 10.3390/nano9121685

**Published:** 2019-11-25

**Authors:** Afeefah Khazi-Syed, Md Tanvir Hasan, Elizabeth Campbell, Roberto Gonzalez-Rodriguez, Anton V. Naumov

**Affiliations:** 1 Department of Physics and Astronomy, Texas Christian University, TCU Box 298840, Fort Worth, TX 76129, USA; afeefahk@mit.edu (A.K.-S.); tanvir.hasan@tcu.edu (M.T.H.); e.sizemore@tcu.edu (E.C.); 2 Department of Chemistry and Biochemistry, Texas Christian University, TCU Box 298860, Fort Worth, TX 76129, USA; r.gonzalezrodriguez@tcu.edu

**Keywords:** carbon nanotubes, antibiotic, methicillin, doxycycline, antibiotic delivery, fluorescence imaging, antibiotic resistance

## Abstract

Although conventional antibiotics have evolved as a staple of modern medicine, increasing antibiotic resistance and the lack of antibiotic efficacy against new bacterial threats is becoming a major medical threat. In this work, we employ single-walled carbon nanotubes (SWCNTs) known to deliver and track therapeutics in mammalian cells via intrinsic near-infrared fluorescence as carriers enhancing antibacterial delivery of doxycycline and methicillin. SWCNTs dispersed in water by antibiotics without the use of toxic bile salt surfactants facilitate efficacy enhancement for both antibiotics against *Staphylococcus epidermidis* strain showing minimal sensitivity to methicillin. Doxycycline to which the strain did not show resistance in complex with SWCNTs provides only minor increase in efficacy, whereas the SWCNTs/methicillin complex yields up to 40-fold efficacy enhancement over antibiotics alone, suggesting that SWCNT-assisted delivery may circumvent antibiotic resistance in that bacterial strain. At the same time SWCNT/antibiotic formulations appear to be less toxic to mammalian cells than antibiotics alone suggesting that nanomaterial platforms may not restrict potential biomedical applications. The improvement in antibacterial performance with SWCNT delivery is tested via 3 independent assays—colony count, MIC (Minimal Inhibitory Concentration) turbidity and disk diffusion, with the statistical significance of the latter verified by ANOVA and Dunnett’s method. The potential mechanism of action is attributed to SWCNT interactions with bacterial cell wall and adherence to the membrane, as substantial association of SWCNT with bacteria is observed—the near-infrared fluorescence microscopy of treated bacteria shows localization of SWCNT fluorescence in bacterial clusters, scanning electron microscopy verifies SWCNT association with bacterial surface, whereas transmission electron microscopy shows individual SWCNT penetration into bacterial cell wall. This work characterizes SWCNTs as novel advantageous antibiotic delivery/imaging agents having the potential to address antibiotic resistance.

## 1. Introduction

Due to the misuse and overuse of conventional antibiotics, resistant infections are on the rise [1]. Those include well-known bacterial strains such as *Staphylococcus aureus* (MRSA) [2], *Streptococcus pneumoniae* [3] and *Mycobacterium tuberculosis* [4]. It is predicted that approximately 10 million people will die from resistant infections by the year 2050 [5], not considering the emergence of new resistant strains. This mortality prediction exceeds that of cancer and diabetes combined. In response to this crisis, new antibiotics are being developed [6,7]; however, offering only a temporary solution and, rather, giving rise to multi-drug resistant infections [8]. With mutated bacterial infections being the foundation for many large-scale health issues including *M. tuberculosis*, MRSA and VRE (Vancomycin-resistant Enterococci) [9,10], the crisis of antibiotic resistance, as well as the need in enhancing antibiotic efficacy becomes a global issue.

To date, very few routes were explored to address the antibiotic resistance including the inhibition of mutation [11], change in the dosing regimen of existing antibiotics [12] and delivery-assisted combination treatments [13,14,15]. However, dosing strategies provide only a temporary solution that delays the formation of resistance, while inhibitory approaches have to be applied to bacteria prior to mutation that renders the antibiotics ineffective. With the few successful delivery-assisted attempts to address the existing antibiotic resistance, current studies still focus more on the development of new antibacterial strategies [16,17,18,19]. In this work, an alternative multimodal approach to the issue at hand is explored. Although, microbial growth inhibition by carbon nanotubes platform was shown before [20], in this work we propose novel non-covalent formulation of existing antibiotics with single-walled carbon nanotubes (SWCNTs) for delivery, imaging, and enhanced antibiotic efficacy.

For the context of this study, the SWCNT-antibiotic dispersions are tested against *Staphylococcus epidermidis*. Because of the increased use of biomaterials in the hospital and clinical environment, *S. epidermidis* has become one of the five most common bacteria to cause nosocomial infections on prosthetic parts, valves, surgical wounds, urinary tract or bone marrow transplants. While already causing nearly one million infections and many deaths per year, *S. epidermidis* has become resistant to a wide scope of antibiotics [21]. Strains of *S. epidermidis* are resistant to methicillin, penicillin, penems, carbapanems, and cephalosporins [22]. With these being the most commonly used antibiotics, an increase in *S. epidermidis* infections becomes a big threat [23].

Biocompatible lipid-based carriers are known to yield high encapsulation efficiency for guest drug molecules [24,25,26,27,28]. On the other hand, SWCNTs offer great promise as antibiotic delivery vehicles due to their unique physical and optical properties. Known for their quasi-one-dimensional structure, SWCNTs have the dimensions suitable for cellular internalization [29,30] show low cytotoxicity when formulated [31] and accumulate partly in actin cytoskeleton but exhibit excretion over time [30]. Additionally, a significant amount of SWCNTs can be loaded into a target cell [32], making them suitable for the delivery of hydrophobic drugs and gene therapies sensitive to degradation in blood. So far, SWCNTs have successfully delivered siRNA to cancer cells [33] and tissues [34,35] and such anticancer drugs as cisplatin, methotrexate, and doxorubicin [36,37,38,39]. SWCNTs also show a potential for antibiotic delivery as multiple antibiotics are known to adsorb well on SWCNT surface [40,41,42] and with covalent attachment improve the efficacy of ciprofloxacin [43]. The mechanism of SWCNT interaction with bacteria is so far unknown and can be further explored with molecular imaging. SWCNTs can be used for that purpose as efficient biomarkers since semiconducting species exhibit near-infrared fluorescence penetrating through the layers of biological tissue due to low tissue absorption/scattering in near-IR [44,45,46]. Such image-guided delivery of antibiotics allowing to track their transport and elucidate SWCNT-mediated mechanism of action has not been explored to date as the covalent attachment of antibiotics quenches SWCNT emission.

In this work, we utilize non-covalent SWCNT antibiotic delivery to enhance drug efficacy and track the transport with intrinsic SWCNT fluorescence, while also aiming to circumvent the antibiotic resistance of the *S. epidermis* showing low Methicillin sensitivity. Antibiotic resistance is based partly on enzymatic degradation of the existing antibiotics or a decreased membrane permeability to those. SWCNTs as delivery vehicles can be well-suited to address both of these issues as they are known to protect delivered gene therapeutics [33,34] from enzymatic degradation and enhance internalization of other drug moieties [47]. Non-covalent delivery also improves the possibility of antibiotic release within bacterial cells. Finally, antibacterial properties of SWCNTs known to disrupt the membrane and/or metabolic processes and morphology of bacteria [48] may serve to the enhancement of antibacterial treatment efficacy. This all suggests that SWCNTs may be highly advantageous delivery vehicles for antibiotic treatment.

## 2. Materials and Methods

### 2.1. Dispersion of SWCNT in Antibiotic Solutions

Concentrated and supersaturated aqueous Doxycycline (20 mg/mL) (purchased from Alfa Aesar) and Methicillin (25 mg/mL) (purchased from Sigma Aldrich) antibiotic suspensions were prepared for SWCNT complexation and further dilution to concentrations used in the antibacterial efficacy studies. Each antibiotic in aqueous suspension was complexed with 500 µg of raw HiPco (Nanointegris batch # HR27-075A) non-covalently via 30 min of ultrasonic bath treatment followed by 20 min ultrasonic tip treatment at 16.5 W of power. Resulting suspensions containing antibiotic-suspended SWCNTs were characterized via absorption spectroscopy and stored at 4 °C with further exposure to 2 min ultrasonic treatment prior to use.

For control experiments, a solution of SWCNTs/DSPE-PEG 5000 was prepared—0.5 mg of SWCNT was added to a 1600 µM solution of DSPE-PEG 5000 (NanoCS) and subjected to the aforementioned ultrasonic dispersion and filtration procedures to yield final SWCNTs/DSPE-PEG-5000 (1,2-distearoyl-sn-glycero-3-phosphoethanolamine-N-[methoxy(polyethylene glycol)-5000]) suspensions.

### 2.2. Characterization of SWCNT-Antibiotic Dispersions

The concentration of all SWCNT suspensions was characterized via absorption spectroscopy. Using standard calibration curve constructed from absorptions of unfiltered SWCNT/antibiotic fractions with known SWCNT amounts we have experimentally derived extinction coefficients at 632 nm for SWCNT dispersed with both drugs (0.015 (µg/mL)^−1^ for SWCNTs/doxycycline and 0.0134 (µg/mL)^−1^ for SWCNTs/methicillin). We further used those to assess the concentration of SWCNTs in centrifuged suspensions.

The concentration of antibiotics in the suspensions of antibiotic/SWCNT hybrids was assessed via deconvoluting absorption spectra of those into components for SWCNTs and antibiotics. SWCNTs/DSPE-PEG 5000 spectra were used as an assessment for SWCNT component and antibiotic standards at known concentrations were used as reference component for antibiotics. This calculation showed w/w ratios of 1:4 for SWCNT/methicillin and 1:5 for SWCNT/doxycycline in stock SWCNT suspensions that were further used throughout this work.

Near-infrared fluorescence of antibiotic/SWCNT suspensions was collected via Nanofluorescence NS2 Nanospecralyzer spectrometer with 637 nm laser excitation for SWCNT antibiotic suspensions after preparation and after a 24 h treatment period. Minimal agitation is applied to ensure no loose aggregation.

### 2.3. Disk Diffusion Assay

*S. epidermidis* (VWR 470176-542) broth of McFarland 0.5 standard (absorption of 0.08 to 0.1) was created with stationary phase culture using Mueller Hilton Broth. This standard stabilized the cell count at an approximate 1 × 10^8^ CFU/mL [49]. Dilution was plated within 15 min of standardization. Following the proper aseptic techniques, 0.2 mL of bacterial broth was placed in the center of prepared agar dish. A sterile bacteria spreader was used to evenly spread the bacteria throughout the plate to create a lawn.

We tested two different dosages of the antibacterial solutions to increase the breadth and reliability of data. Blank sensitivity discs were loaded with 10 µL and 20 µL (based on respective dosage) of stock suspensions and placed onto the surface of the agar using sterile forceps. Discs were impregnated with the test solution dropwise. Five discs were evenly placed equidistant from one another. Before tilting over the Petri dishes, discs were left to dry and gently pressed down to ensure attachment to agar. Once all Petri dishes are prepared, they were turned upside down to prevent surface condensation. Petri dishes are incubated for 24 h at 37 °C, then the zones of inhibition were measured with the inclusion of disk diameter in the measurements. The 24 h time point is chosen based on previous work [50] suggesting a possibility of bacterial growth in antibiotic-resistant strains beyond 20 h and employing this time interval for the assessment of bacterial growth.

### 2.4. Colony Count Assay

Using *S. epidermidis* broth (McFarland 0.5 standard), 100 µL was placed in the center of the agar plate. 100 µL of the respective antibacterial stock solution is added to the center. Bacteria were spread through the Petri dish and the plates were further incubated for 24 h at 37 °C. Pictures of the plates were uploaded onto the OpenCFU software to count the number of colonies grown on the plate. Two plates were prepared for each antibacterial treatment with corresponding controls.

### 2.5. MIC (Minimal Inhibitory Concentration) Turbidity Assay

A serial dilution (using the factor of 2) of antibacterial solutions was conducted in 12-well plates starting with 200 µL of antibacterial solution placed in first well. 850 µL of broth and 50 µL of bacteria in broth were added to each well plate. The solubility of doxycycline and methicillin was 50 and 0.31 mg/mL, respectively according to the manufacturer (Methicillin: Sigma Aldrich; Doxycycline: Alfa Aesar) information. Thus, the antibiotic stock complexed with SWCNTs for all aqueous experiments was diluted to lower concentrations: for doxycycline: 1, 0.5, 0.25, 0.125 and 0 mg/mL and for methicillin are 1.25, 0.625, 0.313, 0.106 and 0 mg/mL. Plates were then incubated at 37 °C for 24 h. The solutions were transferred to cuvettes and their MIC turbidity was measured using a Cary 500 spectrophotometer with the broth used as a baseline. Two wells were prepared for each concentration.

### 2.6. Cytotoxicity Assay

An MTT (3-(4,5-Dimethylthiazol-2-yl)-2,5-diphenyltetrazolium bromide)-based cytotoxicity assay was conducted for 4 samples—doxycycline, SWCNTs/doxycycline, methicillin and SWCNTs/methicillin. Each sample was prepared via serial dilutions at the testing concentrations ranging from 0 to 3.5 µg/mL for doxycycline and SWCNTs dispersed with doxycycline and 0 to 0.25 µg/mL for methicillin and SWCNTs dispersed with methicillin. The absorbance was measured using the FLUOstar Omega microplate reader and was analyzed using Omega software to yield cell viability levels.

### 2.7. Microscopy

We utilized InGaAs near-IR (NIR) camera coupled to hyperspectral fluorescence filter (Photon etc.) to perform fluorescence microscopy of SWCNTs imaged in bacterial cells 24 h after introducing them to bacterial culture. The sample was excited with 637 nm diode laser excitation at 130 mW output power. SWCNTS showed up in the NIR broadband (900–1450 nm) images as bright fluorescent objects. Non-treatment control images were taken for each antibiotic target ensuring no emission in the near-IR. Scanning Electron Microscope (SEM; JEOL-JSM-7100F) was used at 5 kV to image bacterial cells. Samples were prepared by depositing bacteria from the culture onto conductive carbon tape via drop-casting of ~100 µL of SWCNT/antibiotic-treated bacteria in the media. SEM allowed imaging of the outer surface of bacterial cells and extracellular SWCNTs. Transmission Electron Microscopy (TEM; JEOL JEM-2100 TEM) was further utilized to assess the incorporation of SWCNTs into bacteria and the coating of SWCNTs with antibiotic only. Samples for TEM were prepared by drying ~10 µL of either SWCNT/antibiotic suspensions or SWCNT/antibiotic-treated bacterial culture on the carbon-coated 200-mesh copper grid under ambient conditions.

## 3. Results and Discussion

### 3.1. Characterization

Noncovalently complexed SWCNT/antibiotic hybrids were prepared in this work for the first time. Antibiotics are utilized both as a payload and as a surfactant for SWCNTs providing aqueous dispersions stable prior to treatment and over a treatment period showing no substantial SWCNT fluorescence intensity decrease or spectral broadening (Figure S1). Some loose aggregation at 24 h was reversed by mild agitation. The increase in the visible tail of the SWCNT/doxycycline spectrum after 24 h can be attributed to the detachment of some fraction of the antibiotic fluorescing in the visible and no longer quenched by the SWCNTs. Such detachment indicates the possibility of the clearance of the antibiotic from the SWCNT delivery vehicle as those internalize with bacteria rendering antibiotic effective. The coating of antibiotics on SWCNTs has been further assessed by TEM showing substantial surface coverage (Figure S2). Doxycycline and methicillin were specifically chosen for this role due to characteristic hydrophobic regions in their structure some of which are expected to non-covalently bind to SWCNTs via π-stacking. While doxycycline is a tetracycline antibiotic and inhibits reproduction by disrupting protein synthesis, methicillin is a beta-lactam antibiotic that affects the bacteria by interfering with cell wall structure [51,52]. Using two antibiotics from different classifications allows for an understanding of how SWCNTs perform with different modes of action. *S. epidermis* strain used in this work shows no/low response to methicillin suggesting some resistance and thus is intended as a control for the studies.

As SWCNTs alone are insoluble in water, their successful dispersion helps verify their complexation with antibiotics. Characteristic absorption spectra of SWCNTs and antibiotics were used to assess concentrations of those upon complexation. As absorption of both antibiotics is negligible in the visible, the value of absorption at 632 nm is used to determine the amount of SWCNTs (Figure S3a,b). A calibration curve constructed with known concentration of SWCNTs in unfiltered suspensions of SWCNT/antibiotic hybrids allows to determine extinction coefficients for SWCNT/doxycycline and SWCNT/methicillin absorption in the visible to be 0.015 (µg/mL)^−1^ and 0.0134 (µg/mL)^−1^ respectively. Antibiotic concentration in the final dispersion is assessed by deconvoluting spectra of antibiotic/SWCNT hybrids via presenting those as a superposition of those of SWCNT and antibiotic alone at known concentrations, which resulted in the *w*/*w* ratios of ~1:40 for SWCNT/methicillin and ~1:50 and SWCNT/doxycycline aqueous suspensions.

### 3.2. Antibacterial Performance of SWCNT-Antibiotic Dispersions

We use three different antibacterial sensitivity assays to verify the efficacy of SWCNT/antibiotic dispersions. The disk diffusion assay, colony formation assay, and MIC turbidity assay confirm our findings through three different procedures.

#### 3.2.1. Zone Inhibition Assay

**Figure 1:**
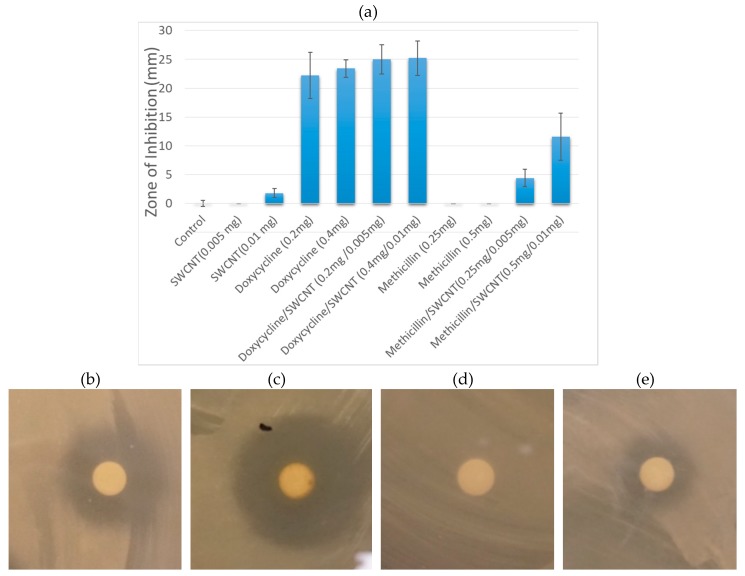
(**a**) Inhibition zones arising from individual components (SWCNT/DSPE-PEG 5000, Doxycycline, Methicillin) and SWCNT/antibiotic hybrids at two different doses delivered to the discs. Images of inhibition zones of *S*. *epidermidis* treated with (**b**) Doxycycline; (**c**) SWCNT/Doxycycline; (**d**) Methicillin; (**e**) SWCNT/ Methicillin.

The antibacterial effects of pure antibiotic solutions are first compared to non-treatment control and vehicle control provided by SWCNT/PEG formulations (Figure 1). Here DSPE-PEG-5000 is used as a biocompatible dispersing agent for SWCNTs to provide stable aqueous dispersions without the use of antibiotics.

In this formulation, SWCNTs show little to no antibacterial effect, which is supportive of their sole role as drug delivery/imaging agents providing no interference with the antibacterial efficacy of the payload. Among the antibiotics, doxycycline is significantly more effective at both 0.2 and 0.4 mg doses, whereas methicillin shows no antibiotic activity with inhibition levels same as non-treatment control (Figure 1). Since strains of *S. epidermis* are known to show antibiotic resistance and several are specifically resistant to methicillin [53]. The complete lack of inhibition response with this antibiotic for colony formation and disc diffusion assays likely indicates a resistant behavior of the current strain to methicillin. At the same time no resistance to doxycycline has been observed.

Concentrations of antibiotics in complexes with SWCNTs were further chosen to mimic those of unformulated antibiotics. SWCNT/antibiotic dispersions show substantial improvement in the inhibition response for both antibiotics. SWCNT/Doxycycline complexes become slightly more effective than Doxycycline alone (8% improvement), whereas SWCNT/Methicillin hybrids exhibits a drastic improvement—an increase in efficacy from no observable bacterial inhibition to the 50% of the inhibition response of doxycycline. Because *S. epidermidis* in this test initially shows no response to methicillin, the appearance of significant antibacterial response suggests that SWCNT delivery may bypass such antibiotic resistance. While there are a number of theories regarding the mechanisms of internalization of carbon nanotubes into prokaryotic cells, [54,55] many are suggesting effective cell wall penetration. Additionally, SWCNTs are known to introduce substantial disruption of the bacterial cell wall [56,57,58] and facilitate plant cell wall penetration of the payload that alone does not show successful internalization [59]. These mechanisms of nanotube interactions with cell walls and membranes at higher concentrations may lead to antibacterial effects [31,60]. Thus, considering that methicillin’s mechanism of action is based on the inhibition of cell wall synthesis it is plausible that SWCNT/cell wall interaction may prevent the recognition of antibiotic by the resistant bacteria and facilitate its delivery inside the cell wall where methicillin may successfully perform its primary function.

#### 3.2.2. Statistical Analysis

The statistical analysis performed in this work confirms the significance of the observed results for diffusion discs performed at different amounts of SWCNT/antibiotic complex loaded onto discs. Data analysis is performed using JMP software utilizing ANOVA (Analysis of Variance) to provide statistical information to understand the predominant effects and significance of differences in data. In performing an ANOVA on a dataset, we make a null hypothesis, stating that the average means of the various treatments are the same. When the p-value of the analysis is lower than the confidence level chosen, the hypothesis is proven false and a significant difference and variation is detected [61].

The dataset is analyzed using a comparison of means function by assigning a mean of the control group and comparing it to the means of each of the treatment groups. The degree of overlap of the circles in the graphic represents their significant similarity or difference, whereas the size is proportional to the corresponding variance [31].

**Figure 2:**
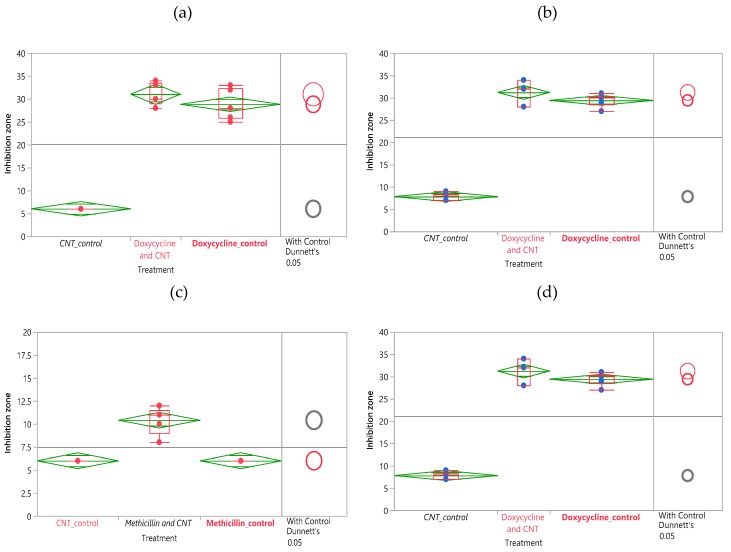
ANOVA and Dunnet’s method statistical analysis of doxycycline disk diffusion data for (**a**) 0.5 mg SWCNT, 2 mg antibiotic loaded (**b**) 1 mg SWCNT, 4 mg antibiotic loaded. ANOVA and Dunnet’s method statistical analysis of methicillin disk diffusion data for (**c**) 0.5 mg SWCNT, 2.5 mg antibiotic loaded (**d**) 1 mg SWCNT, 5 mg antibiotic loaded.

For comparison of antibiotic effects of SWCNT/Doxycycline to the free antibiotic (Figure 2a,b), the differences in data are significant. For both dosages of doxycycline, the R-square is fairly high (~0.97) and the Prob >F is low (<0.0001), indicating a considerably good fit of the data. Based on the Control Dunnett’s method, the difference between the control (antibiotic alone) and SWCNT/antibiotic hybrids is insignificant for 2 mg dosage but is considered significant for 4 mg dosage. Overall, we can infer that the complexes of SWCNT/doxycycline exhibit marginally better activity than antibiotic alone. Unlike in the case of doxycycline, statistical analysis shows a significant improvement in antibacterial efficacy for SWCNT/methicillin hybrids. R-squares are relatively high (~0.9 and 0.8) and prob>F are low (<0.0001), indicating a good fit of the data. The Control Dunnet’s circles for both doses (Figure 2b,c) show that the difference between control and treatment is significant and SWCNT/methicillin complex is statistically much more efficacious than methicillin alone.

#### 3.2.3. Colony Formation Assay

**Figure 3:**
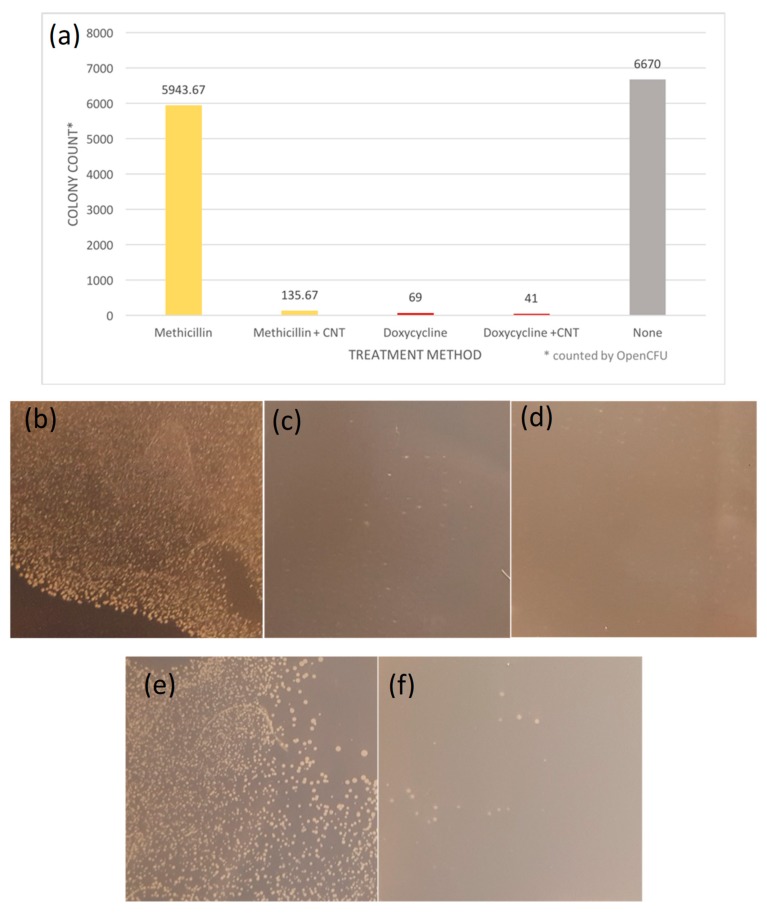
(**a**) Colony Formation Unit assay of antibiotics and antibiotic-SWCNT dispersion. Colonies on each plate counted with OpenCFU software. (**b**) *S*. *epidermidis* control (**c**) *S*. *epidermidis* treated with Doxycycline (**d**) *S*. *epidermidis* treated with Doxycycline-SWCNT dispersion (**e**) *S*. *epidermidis* treated with Methicillin (**f**) *S*. *epidermidis* treated with Methicillin-SWCNT dispersion.

The results from the colony formation assay support that the efficacy of SWCNT/antibiotic hybrids can be improved by the SWCNT delivery. SWCNT/Doxycycline formulation provides only slightly lower colony counts with only 68% improvement over the control, so we consider it together with control both similarly effective against *S. epidermis*. The colony count for methicillin alone is similar to the control, as expected, whereas that for SWCNTs/methicillin is suppressed 40-fold (4000% improvement) showing significant observable (Figure 3f) decrease in the number of colonies. Such a drastic increase in efficacy for SWCNT-delivered methicillin with corresponding only minor improvement for already effective doxycycline can be likely attributed to the bypassing of antibiotic resistance via SWCNT delivery.

#### 3.2.4. MIC turbidity Assay

**Figure 4:**
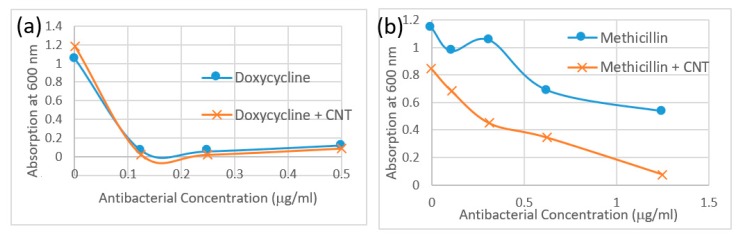
Minimum inhibitory concentration (MIC) turbidity assay absorption data at 600 nm for (**a**) Doxycycline (blue circles) and SWCNT/Doxycycline (red squares). (**b**) Methicillin (blue circles) and SWCNT/Methicillin (red squares).

The MIC turbidity assay performed with SWCNT/antibiotic formulations in bacterial media assessed scattering from turbid samples proportional to the bacterial concentration in suspension. Relative scattering is assessed by the magnitude of scattering background in absorption spectra sampled at 600 nm. Due to the low concentrations of antibiotics and SWCNT used in this study, SWCNT absorption does not interfere with MIC turbidity measurements. The absorption data in Figure 4a,b is therefore presented as a Minimum Inhibitory Concentration Test. Rather than using a MIC value however, the overall MIC turbidity curve is utilized to assess the amount of bacteria remaining in the respective suspensions. As consistent with findings of two previous methods, bacteria treated with SWCNT/methicillin complexes exhibit a lower MIC turbidity than the same doses of methicillin alone. Although antibiotic control for this test shows some bacterial inhibition, a significant improvement upon SWCNT complexation is noted. Doxycycline/SWCNTs hybrids yield slightly lower MIC turbidity in bacterial media as opposed to antibiotic alone as opposed to significant improvement for Methicillin.

As seen in all of the bacterial sensitivity assays, the complexation of antibiotics with SWCNTs increases the efficacy of the treatment when compared to antibiotic alone. SWCNT in this role may act as efficient drug carriers or, potentially, enhance the effect of antibiotics in a combination treatment. While the improvement of doxycycline efficacy via the dispersion with SWCNTs is minimal, SWCNT/Methicillin complexes become far more efficacious bypassing the antibiotic resistance to methicillin.

### 3.3. Cytotoxicity

**Figure 5:**
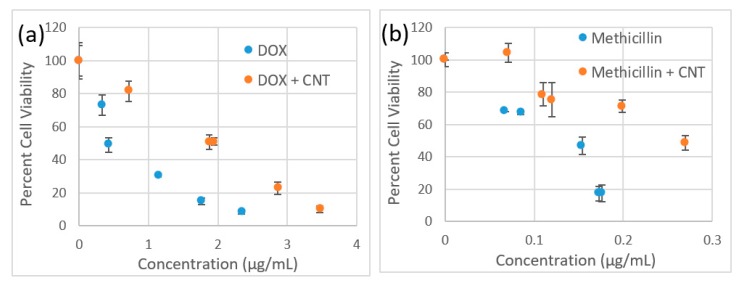
Cytotoxicity assay in HeLa cells comparing response to (**a**) Doxycycline (blue circles) vs. SWCNT/Doxycycline (red circles). (**b**) Methicillin (blue circles) vs. SWCNT/Methicillin (red circles).

We verify that the observed effect is not due to inherent SWCNT toxicity in complexation with methicillin by a separate MTT cytotoxicity assay in HeLa cells (Figure 5). Considering that *S. epidermis* thrives in epithelial microflora, we have chosen a HeLa cell line with epithelial morphology in which SWCNT internalization and interaction were best characterized [62,63,64]. For both Doxycycline and Methicillin SWCNT/antibiotic complexes at the same antibiotic dose show less inherent toxicity to HeLa cells than antibiotics alone indicating no toxic effect from SWCNTs. A lower cytotoxic response of SWCNT-complexed antibiotics also suggests that SWCNTs do not add to the toxic profile of the formulation in mammalian cells and would not restrict its potential biomedical applications.

### 3.4. Imaging

**Figure 6:**
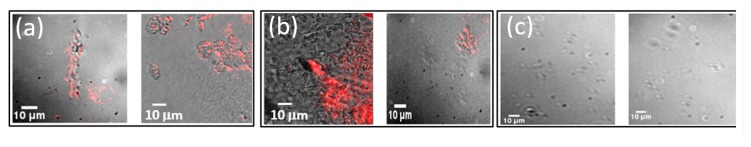
Near-infrared (NIR) Fluorescence imaging of SWCNT emission in bacterial cells subject to (**a**) SWCNT/Methicillin treatment; (**b**) SWCNT/Doxycycline treatment; (**c**) Non-treatment control.

We utilize the inherent emission of SWCNTs in the near-IR to track and image those in bacterial cells collected from the bacterial culture around the discs in the disc diffusion assay to verify cell internalization. Control bacteria were imaged together with the ones subject to SWCNT/methicillin and SWCNT/doxycycline treatments loaded on the discs. As biological autofluorescence background is minimal in the near-IR, we expect SWCNTs to be the major emissive species. In accordance with this, control cells show no observable emission whereas microscopy images of bacterial cells recovered in the vicinity of the discs with SWCNTs/methicillin and SWCNTs/doxycycline show bright near-IR SWCNT emission in the clusters bacterial cells (Figure 6). The highest signal intensities were found surrounding cells or cell clusters suggesting possible association with bacterial cell wall. Extracellular SWCNT emission appears to be rare indicating preferential interaction of SWCNT/antibiotic hybrids with bacteria.

**Figure 7:**
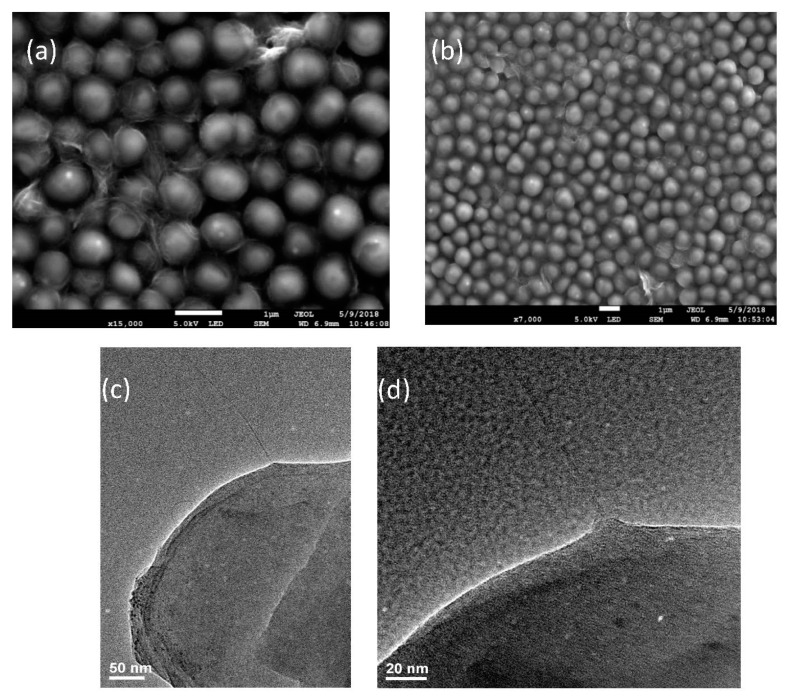
Electron Microscopy (**a**,**b**) Scanning electron microscopy (SEM) images of bacterial cells subject to SWCNT/methicillin hybrids, (**c**,**d**) Transmission electron microscopy (TEM) images of SWCNTs penetrating bacterial cell wall in cultures subject to SWCNT/methicillin.

Considering resolution limitations of fluorescence imaging of small micrometer-sized bacterial cells we verify these findings by the higher resolution SEM imaging of bacteria subject to SWCNT/methicillin hybrids. In SEM images showing the outer surface of the bacteria, SWCNT are clearly observed associating with the wall of bacterial cells in large quantities (Figure 7a,b) with some incidences of cell wall interaction caught in the image. This is further confirmed by TEM images of bacterial culture subjected to SWCNT/methicillin. High resolution of TEM allows looking at individual bacteria at a time showing several cases of SWCNT penetration into the cell wall (Figure 7c,d).

This preferential accumulation of SWCNTs with further internalization may lead to the enhanced delivery and somewhat improved antibiotic effect observed for some experiments with SWCNT/doxycycline. Further works will involve the assessment of internalization and dynamics with FLIM imaging. Considering that the mechanism of action of methicillin is based on the inhibition of cell wall synthesis, SWCNT-assisted penetration into cell wall may significantly improve its efficacy and potentially bypass mechanisms of antibiotic resistance. SWCNTs known to associate well with cell membranes [48,65] are expected to deliver methicillin to the outer surface of the membrane while protecting it from methicillin-hydrolyzing β-lactamase [66]. This does not address the overexpression of or hampered binding of methicillin to mutated PBP proteins (PBP2A) in *S. epidermis* [67] or other strains [52]. However, similarly to membrane-adherent Triton X-100 [68], SWCNT delivery may still improve methicillin susceptibility partially circumventing antibiotic resistance or destabilize the cell wall. Experimental results observed here suggest that SWCNTs acting as delivery vehicles with no inherent antibacterial activity provide an alternative route to cell wall internalization and improvement of methicillin efficacy.

Despite the in vitro focus of this work considering potential future in vivo applications of SWCNT/antibiotic conjugates, we anticipate only minimal toxicity and/or immunogenicity observed for a number of SWCNT formulations [69,70,71,72]. However, the response to SWCNTs is highly coating-dependent and for some formulations, adverse immune responses were detected [73,74,75]. Thus, considering the nature of *S. epidermis* bacteria, a safer topical administration of the antibiotic/SWCNT conjugates can be considered.

## 4. Conclusions

This work, for the first time, explores the joint delivery and imaging of antibiotics by single-walled carbon nanotubes. SWCNTs dispersed in water with doxycycline and methicillin non-covalently attached to their surface act as drug delivery vehicles facilitating the improved antibacterial effect in *Staphylococcus epidermidis*. In three different sensitivity assays performed in this work, the advantages of a SWCNT/antibiotic therapy are apparent. SWCNTs facilitate preferential bacterial accumulation and internalization enhancing antibacterial effect for methicillin with marginal improvement for doxycycline. SWCNT delivery yields a 40-fold (4000%) improvement in bacterial colony inhibition for the SWCNT complex with methicillin, to which *S*. *epidermidis* initially shows resistant behavior in our assays. These results confirmed by statistically significant findings from disc diffusion and a general trend provided by the MIC turbidity assays suggest that whereas for doxycycline positive variations in efficacy can be explained by potentially increased uptake facilitated by SWCNT delivery; SWCNT/methicillin complexes likely circumvent the antibiotic resistance of *S*. *epidermidis.* SWCNTs are expected to penetrate bacterial cell walls delivering methicillin and protecting it against degradation. Based on the reported interaction of SWCNTs with the cell wall and cellular membrane we consider that direct transport of the antibiotic by the SWCNTs may introduce cell wall disruption and/or facilitate enhanced delivery and increasing susceptibility of bacteria to methicillin.

These internalization-based hypotheses are supported by SWCNT fluorescence imaging within bacterial cell culture subject to SWCNT/antibiotic treatment indicating substantial SWCNT fluorescence signal originating from bacteria rather than extracellular environment. SEM images confirm the association of SWCNTs with the cell wall of bacteria, whereas TEM verifies successful cell wall internalization by SWCNTs. In this work, SWCNT acts as effective multifunctional antibiotic delivery/imaging agents with the potential to circumvent antibiotic resistance. As methicillin is one of the more widely known antibiotics for developing resistance, its activation through noncovalent hybridization with SWCNTs offers an alternative potential approach to the antibiotic resistance issue. It may further provide a chance to reduce the dose, reuse and recycle the existing antibiotics for the treatment of the new resistant bacterial epidemics.

## Supplementary Materials

The following are available online at https://www.mdpi.com/2079-4991/9/12/1685/s1, Figure S1: Fluorescence spectra of SWCNTs/Doxycycline and (SWCNTs/Methicillin suspensions as prepared and a 24 h after, Figure S2: TEM images of SWCNTs dispersed with doxycycline and methicillin showing antibiotic coating on nanotubes, Figure S3: Absorption spectra of SWCNTs/DSPE-PEG 5000 and antibiotics alone; absorption spectra of complexed SWCNTs and antibiotics, Figure S4: Petri dish pictures for Colony Formation Unit Assay: *S. epidermidis* control, *S. epidermidis* treated with Doxycycline, *S. epidermidis* treated with Doxycycline-SWCNT dispersion, *S. epidermidis* treated with Methicillin, *S. epidermidis* treated with Methicillin-SWCNT dispersion.
