# Supplementary material for: Single-Walled Carbon Nanotube-Assisted Antibiotic Delivery and Imaging in S. epidermidis Strains Addressing Antibiotic Resistance
Source: Nanomaterials (Basel). 2019 Nov 25;9(12):1685. doi: 10.3390/nano9121685 (PMC6955706; doi:10.3390/nano9121685)
Supplement: Supplementary file 1 [file nanomaterials-09-01685-s001.pdf]

nanomaterials

Article

Single-Walled Carbon Nanotube-Assisted Antibiotic Delivery and Imaging in *S. epidermidis* Strains Addressing Antibiotic Resistance

Afeefah Khazi-Syed [1,†], Md Tanvir Hasan [1], Elizabeth Campbell [1], Roberto Gonzalez-Rodriguez [2] and Anton V. Naumov [1,*]

[1] Department of Physics and Astronomy, Texas Christian University, TCU Box 298840, Fort Worth, TX 76129, USA; afeefahk@mit.edu (A.K.-S.); tanvir.hasan@tcu.edu (M.T.H.); e.sizemore@tcu.edu (E.C.)
[2] Department of Chemistry and Biochemistry, Texas Christian University, TCU Box 298860, Fort Worth, TX 76129, USA; r.gonzalezrodriguez@tcu.edu
* Correspondence: a.naumov@tcu.edu
† Current address: Massachusetts Institute of Technology, Cambridge, MA 02139, USA.

Supporting Information

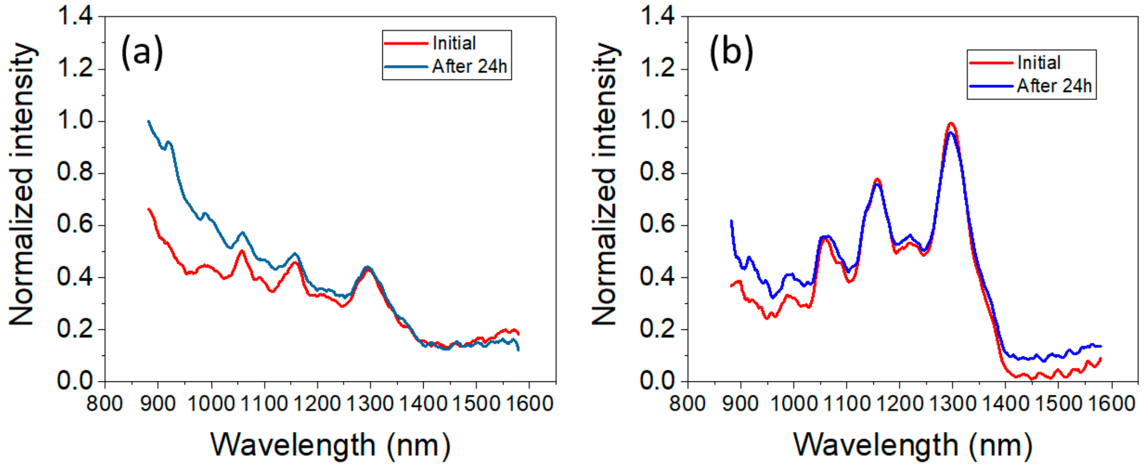

Figure S1. Fluorescence spectra of (a) SWCNTs/Doxycycline and (b) SWCNTs/Methicillin suspensions as prepared (red line) and a 24 h after (blue line)

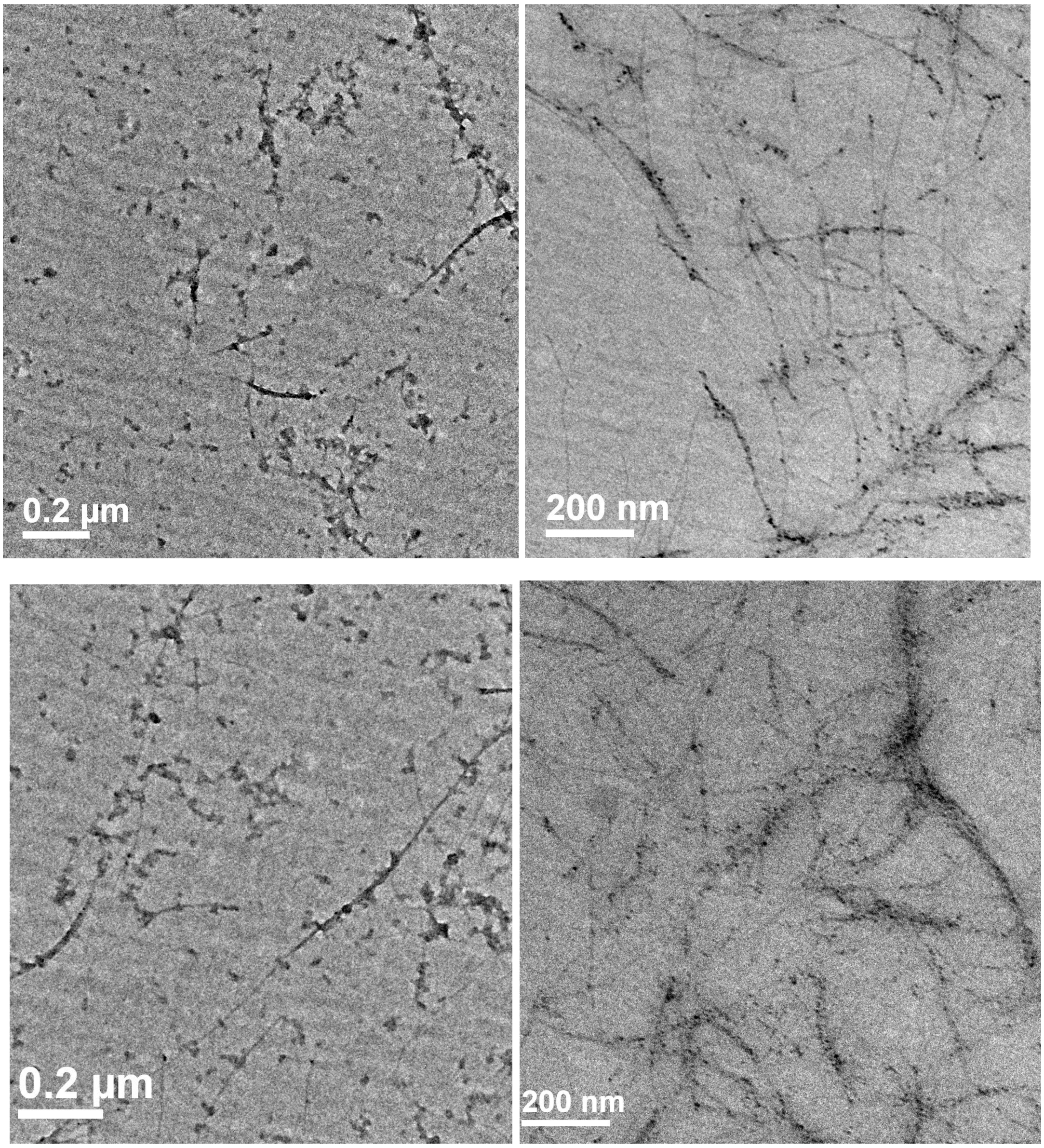

Figure S2. TEM images of SWCNTs dispersed with doxycycline (left panel) and methicillin (right panel) showing antibiotic coating on nanotubes.

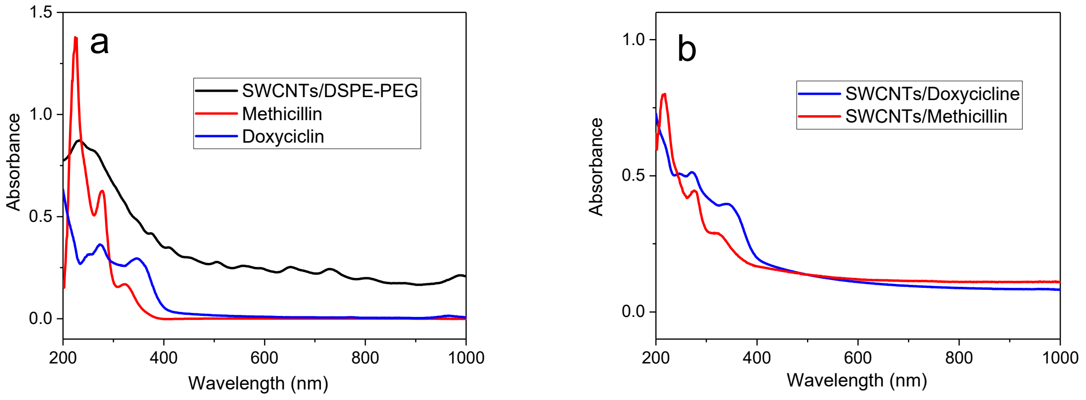

Figure S3. (a) Absorption spectra of SWCNTs/DSPE-PEG 5000 and antibiotics alone. (b) Absorption spectra of complexed SWCNTs and antibiotics.

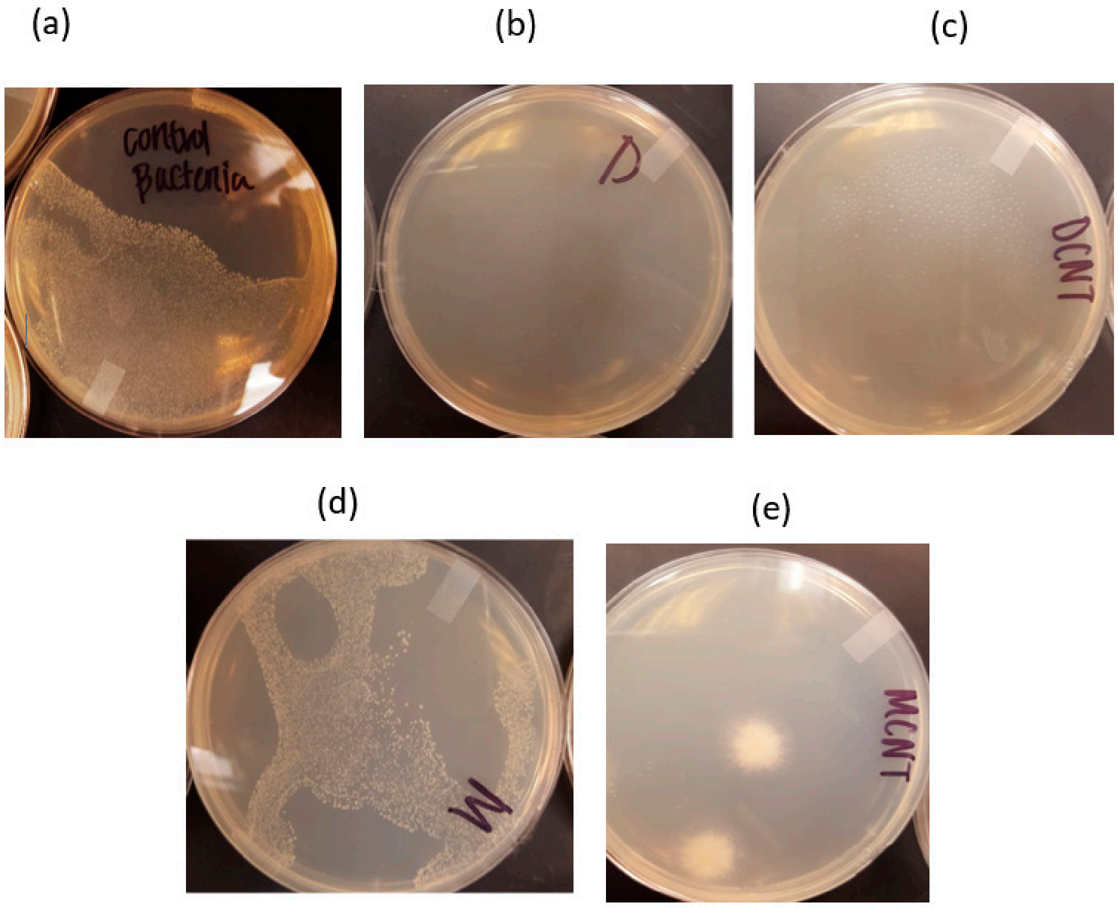

Figure S4 – Petri dish pictures for Colony Formation Unit Assay: (a) S. *epidermidis* control (b) S. *epidermidis* treated with Doxycycline (c) S. *epidermidis* treated with Doxycycline-SWCNT dispersion (d) S. *epidermidis* treated with Methicillin (e) S. *epidermidis* treated with Methicillin-SWCNT dispersion
